# Nutcracker syndrome caused by an enlarged splenic artery in a patient with cirrhosis: a case report

**DOI:** 10.11604/pamj.2025.51.9.47200

**Published:** 2025-05-08

**Authors:** Abdellatif Outrah, Oumaima Tounsi, Youssef Bouktib, Ayoub El Hajjami, Badr Boutakioute, Mariem Ouali Idrissi, Najat Cherif Idrissi El Ganouni

**Affiliations:** 1 *Service de Radiologie, Hôpital* Arrazi CHU Mohamed VI, Marrakech, Maroc

**Keywords:** Nutcracker syndrome, splenic artery, liver cirrhosis, renal vein compression, case report

## Abstract

Nutcracker syndrome is a rare condition caused by the compression of the left renal vein. We present the case of a 40-year-old woman with cirrhosis and splenomegaly, who developed nutcracker syndrome secondary to an enlarged splenic artery compressing the left renal vein. This case highlights the importance of considering unusual anatomical variations in cirrhotic patients presenting with flank pain. Early diagnosis can guide appropriate management and avoid complications.

## Introduction

Nutcracker syndrome refers to symptomatic extrinsic compression of the left renal vein, most commonly between the superior mesenteric artery and the abdominal aorta, presenting with left flank pain and hematuria [1]. Other causes include compressive lymphadenopathy, malignancy, pregnancy, or vascular anomalies [1]. The splenic artery as a cause has not been previously reported [2]. We report a case of nutcracker syndrome caused by splenic artery enlargement in a cirrhotic patient.

## Patient and observation

**Patient information:** a 40-year-old female with a history of liver cirrhosis and splenomegaly presented with recurrent left flank pain, particularly after physical exertion. She reported a childhood history of geophagia.

**Timeline of current episode:** the patient experienced intermittent flank pain over several months, leading to imaging workup after symptoms worsened.

**Clinical findings:** she was alert and oriented. Examination revealed no skin discoloration. Vital signs were normal, and body mass index (BMI) was 22. A urine strip test was positive for hematuria.

**Figure 1:**
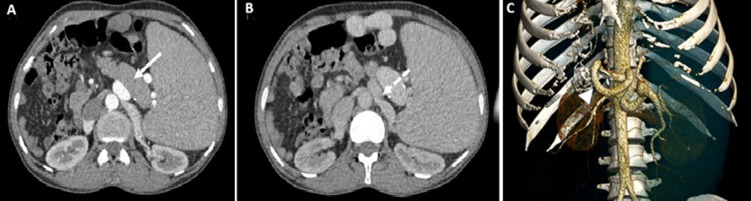
computed tomography (CT) scan and 3D reconstruction demonstrating nutcracker syndrome: A,B) axial CT views and 3D reconstruction; C) an enlarged and serpentine splenic artery (solid arrow), measuring 13mm at its most dilated point; the artery compresses the left renal vein (dotted arrow) against the abdominal aorta, producing a 'beak sign; the hilar-to-compression diameter ratio is 7

**Figure 2:**
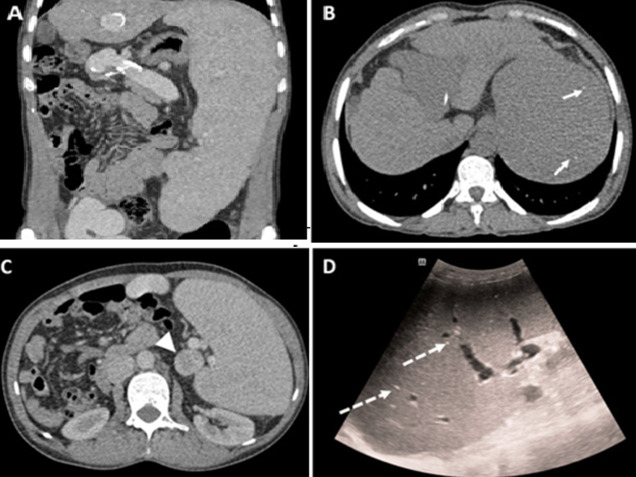
splenic parenchymal findings and accessory spleen identification: A) coronal; B,C) axial CT views along with ultrasound; D) massive splenomegaly (27cm) with multiple hyperdense foci (short arrows) consistent with Gamna-Gandy bodies; a peri-hilar nodular formation (triangle) resembling splenic tissue suggests an accessory spleen; hyperechoic foci with posterior acoustic shadowing are visible on ultrasound (dotted arrows)

**Figure 3:**
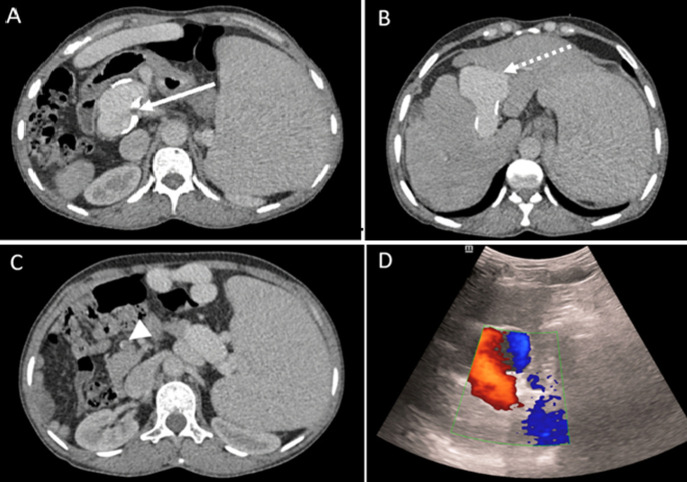
portal venous system abnormalities and thrombosis; A-C) axial CT images showing a dilated portal vein with parietal calcifications; A) a fusiform aneurysm in its intrahepatic segment; B) thrombosis of the superior mesenteric vein; C) Doppler ultrasound; D) a hepatofugal and bidirectional flow pattern

**Figure 4:**
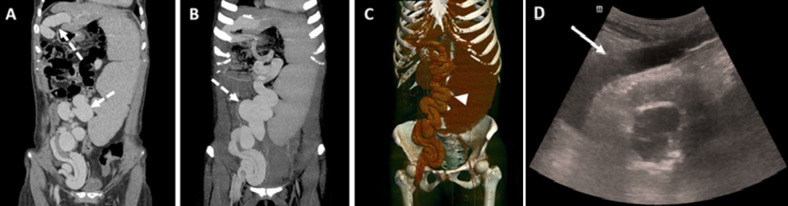
recanalization of abdominal wall veins in portal hypertension: A,B) coronal computed tomography; C) 3D reconstruction, and ultrasound; D) ultrasound showing the patency and flow within the umbilical vein with prominent umbilical and inferior epigastric veins (dotted arrows), measuring 23mm and 31mm respectively, consistent with Cruveilhier-Baumgarten syndrome

**Diagnostic assessment:** abdominal computed tomography (CT) revealed a large, serpentine splenic artery measuring 13mm in diameter, compressing the left renal vein against the abdominal aorta, producing a “beak sign,” and a hilar-to-compression diameter ratio of 7. Panel C highlights the unusual trajectory of the splenic artery occupying the left flank (triangle) hyperdense foci (short arrows) consistent with Gamna-Gandy bodies; a peri-hilar nodular formation (triangle) resembling splenic tissue suggests an accessory spleen; hyperechoic foci with posterior acoustic shadowing are visible on ultrasound (dotted arrows)

(Figure 1). Additional findings included massive splenomegaly (27cm) with multiple hyperdense foci in the parenchyma consistent with Gamna-Gandy bodies, and a peri-hilar nodular formation suggestive of an accessory spleen (Figure 2). A dilated portal vein with parietal calcifications and a fusiform aneurysm in its intrahepatic segment was also observed, complicated by thrombosis of the superior mesenteric vein (Figure 3). Recanalized umbilical and inferior epigastric veins were seen, consistent with Cruveilhier-Baumgarten syndrome (Figure 4).

**Diagnosis:** nutcracker syndrome due to compression of the left renal vein by an enlarged splenic artery.

**Interventions:** given the patient's liver failure and hypoalbuminemia, surgical options were not considered. Long-term surveillance and palliative treatment were initiated.

**Follow-up and outcome of interventions:** the patient continues under clinical observation with symptom monitoring.

**Patient perspective:** the patient was satisfied with her care and expressed regret over her childhood geophagia.

**Informed consent:** the patient provided consent for publication of this case report.

## Discussion

Nutcracker syndrome is a rare vascular compression disorder characterized by entrapment of the left renal vein, most commonly between the abdominal aorta and the superior mesenteric artery, resulting in impaired renal venous outflow and increased venous pressure [3,4]. The condition is more prevalent in women and young adults [4]. While aorto-mesenteric compression is the classic mechanism, various atypical causes have been documented in the literature, including retroperitoneal masses, severe spinal lordosis, pregnancy, and rapid weight loss [3]. Vascular compression by arteries other than the superior mesenteric artery is extremely rare, and to our knowledge, this is the first reported case of left renal vein compression by an enlarged splenic artery. The clinical presentation of nutcracker syndrome is heterogeneous and can include symptoms such as persistent or intermittent left flank pain, microscopic or macroscopic hematuria, orthostatic proteinuria, and pelvic or gonadal vein varices. In men, varicoceles may develop, whereas women may suffer from pelvic congestion syndrome [5]. These symptoms arise due to venous hypertension and collateral venous engorgement secondary to renal vein compression [5]. If left untreated, patients are at risk for progressive renal dysfunction and complications such as renal vein thrombosis, venous aneurysms, or embolization [5]. Radiologic imaging is essential for diagnosing nutcracker syndrome [5].

Doppler ultrasound may demonstrate increased peak velocity at the site of compression and prestenotic dilation. Multidetector computed tomography (CT) and magnetic resonance imaging (MRI) are valuable in visualizing the anatomy and assessing compression severity [6]. Computed tomography findings suggestive of the syndrome include the presence of a beak sign and a hilar-to-aorto-mesenteric diameter ratio greater than 4.9 [5,6]. Invasive techniques such as retrograde venography and intravascular ultrasound may be reserved for equivocal or complex cases [1]. Management of nutcracker syndrome depends on symptom severity [4]. Mild or asymptomatic cases are often managed conservatively with observation [4]. In symptomatic cases with significant hematuria or pain, treatment options include surgical transposition of the left renal vein, endovascular stenting, or renal auto-transplantation [5]. The choice of intervention should be individualized, particularly in patients with underlying comorbidities [4,5]. In this case, we describe a 40-year-old woman with liver cirrhosis, massive splenomegaly, and portal hypertension who presented with recurrent left flank pain. Imaging revealed a markedly dilated and serpentine splenic artery measuring 13mm at its widest point, compressing the left renal vein against the aorta, resulting in nutcracker syndrome. Associated findings included a large spleen with Gamna-Gandy bodies, a fusiform aneurysm of the portal vein, thrombosis of the superior mesenteric vein, and extensive venous collateralization consistent with Cruveilhier-Baumgarten syndrome. Given her hypoalbuminemia and the risk associated with invasive procedures in a decompensated cirrhotic patient, a conservative treatment plan was chosen with long-term clinical surveillance. This case highlights the importance of considering non-classical anatomical causes in the diagnosis of nutcracker syndrome. In cirrhotic patients with complex vascular alterations, unusual presentations should be interpreted cautiously using high-resolution imaging modalities. Timely identification of such anomalies is essential to guide appropriate management and avoid complications such as renal thrombosis or hemorrhagic events.

## Conclusion

This case illustrates an atypical etiology of nutcracker syndrome in a cirrhotic patient. Recognition of unusual vascular anomalies is critical when evaluating unexplained flank pain. Radiologists and clinicians should maintain a high index of suspicion, especially in complex patients, to enable timely diagnosis and management.
